# P-1872. Establishing a Quality Incentive for ID Providers by Leveraging the Standardized Antimicrobial Administration Ratio (SAAR)

**DOI:** 10.1093/ofid/ofaf695.2041

**Published:** 2026-01-11

**Authors:** Lea Edwards, Lou Ann Bruno-Murtha

**Affiliations:** Cambridge Health Alliance, Medford, MA; Cambridge Health Alliance, Medford, MA

## Abstract

**Background:**

Productivity incentives (PI) based on RVUs for infectious diseases (ID) providers fail to recognize much of the quality and value we provide. This may contribute to provider dissatisfaction and burnout. Quality incentives (QI) are more appropriate, but have not been adopted by hospitals or physician organizations (PO). Cambridge Health Alliance, a mission-driven safety-net organization, first recognized and awarded a QI to ID in FY25 for an antibiotic stewardship commitment.

**Figure:**
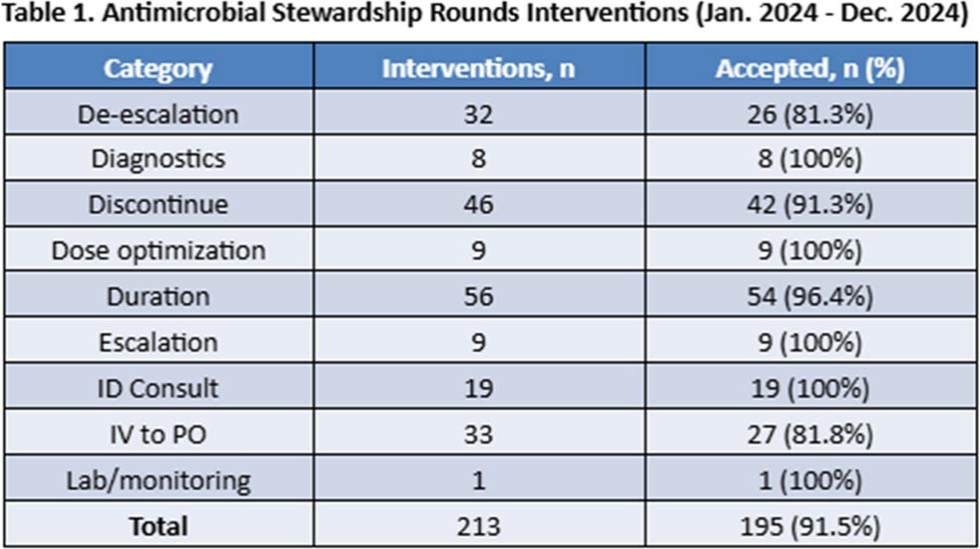

**Figure:**
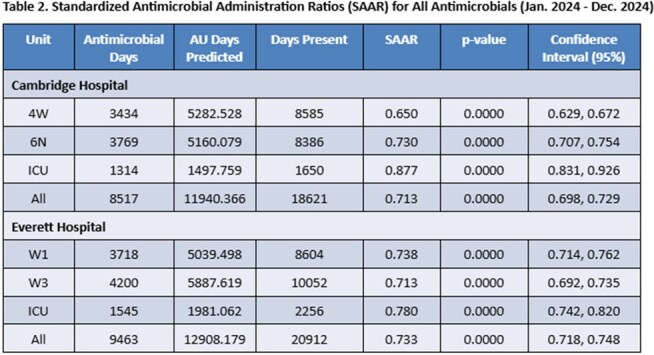

**Methods:**

We utilized the risk-adjusted standardized antimicrobial administration ratio (SAAR), with a target of < 1 as a metric for a QI. Prior data demonstrated we could achieve this goal using our established program of weekly stewardship rounds on medical-surgical units and in ICUs at our two community-based teaching hospitals. Rounds are led by 1 of 4 attendings and an ID clinical pharmacist (CP). A convenience sample of recommendations, intervention categories and acceptance rate was maintained by our CP in 2024. (Table 1).

The ID Division Chief met with the CMO in 2023 to begin the conversation and the Chief of the PO in 2024 to execute the proposal. The Department of Medicine (DOM) leadership was engaged to facilitate a formal approval.

**Results:**

A $40,000.00 quality incentive was granted to the ID Division for FY25 for an achieved overall SAAR < 1. Based on 2024 data (Table 2), we are confident this QI will be allocated and distributed to Division staff at the end of the fiscal year.

**Conclusion:**

This QI may be an opportunity for other ID Divisions to improve compensation. PI fall short in ID where it is challenging to achieve RVU thresholds, particularly in safety-net organizations. The proven value ID provides to patients, staff and organizations requires better monetary remuneration in order to be attractive to future trainees. This is the first QI program established in the DOM.

**Disclosures:**

All Authors: No reported disclosures

